# Supplementary figures and images for: Within-host competition drives energy allocation trade-offs in an insect parasitoid
Source: PeerJ. 2020 Apr 21;8:e8810. doi: 10.7717/peerj.8810 (PMC7182028; doi:10.7717/peerj.8810)

Sex    • Female    ▲ Male

**a**

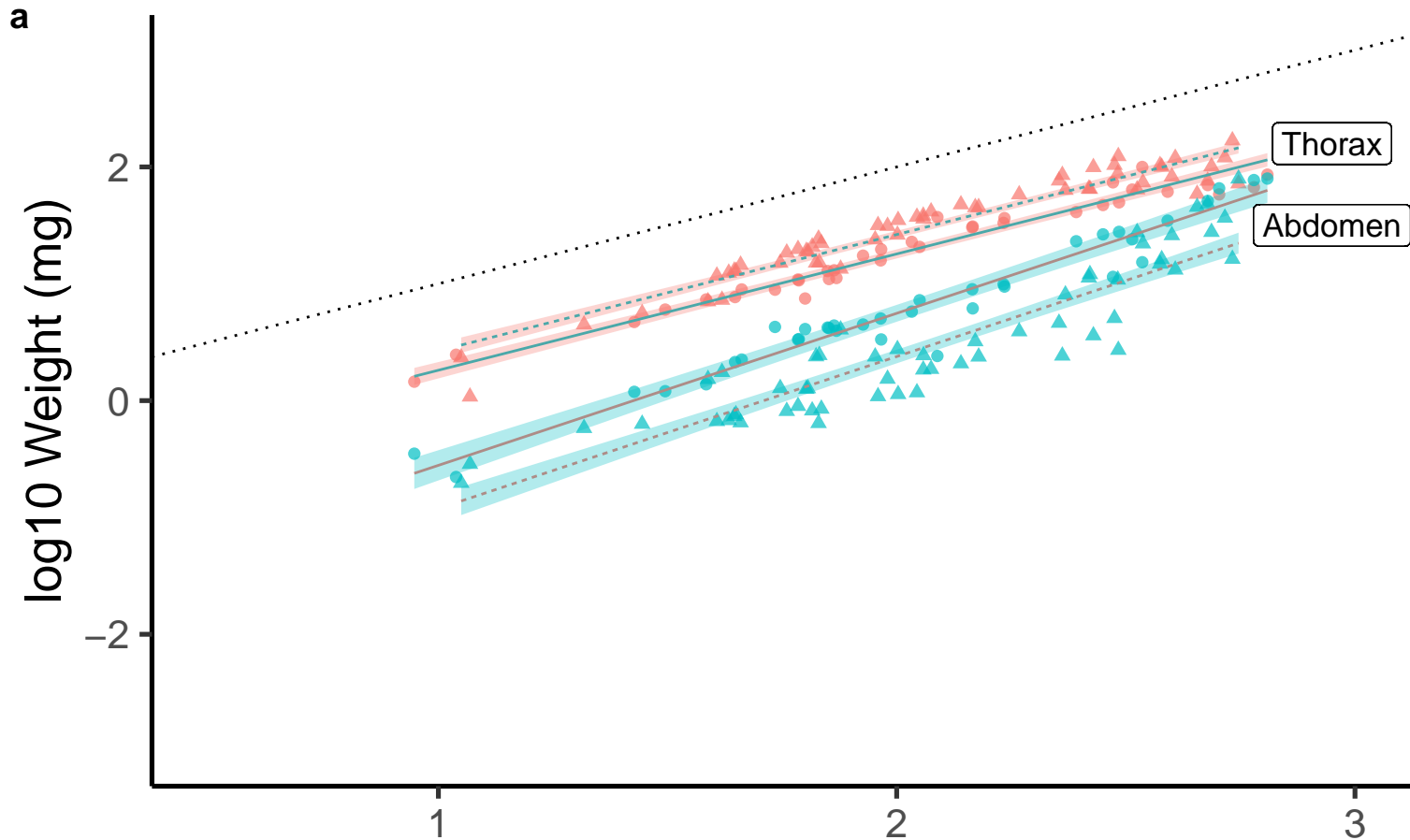

**b**

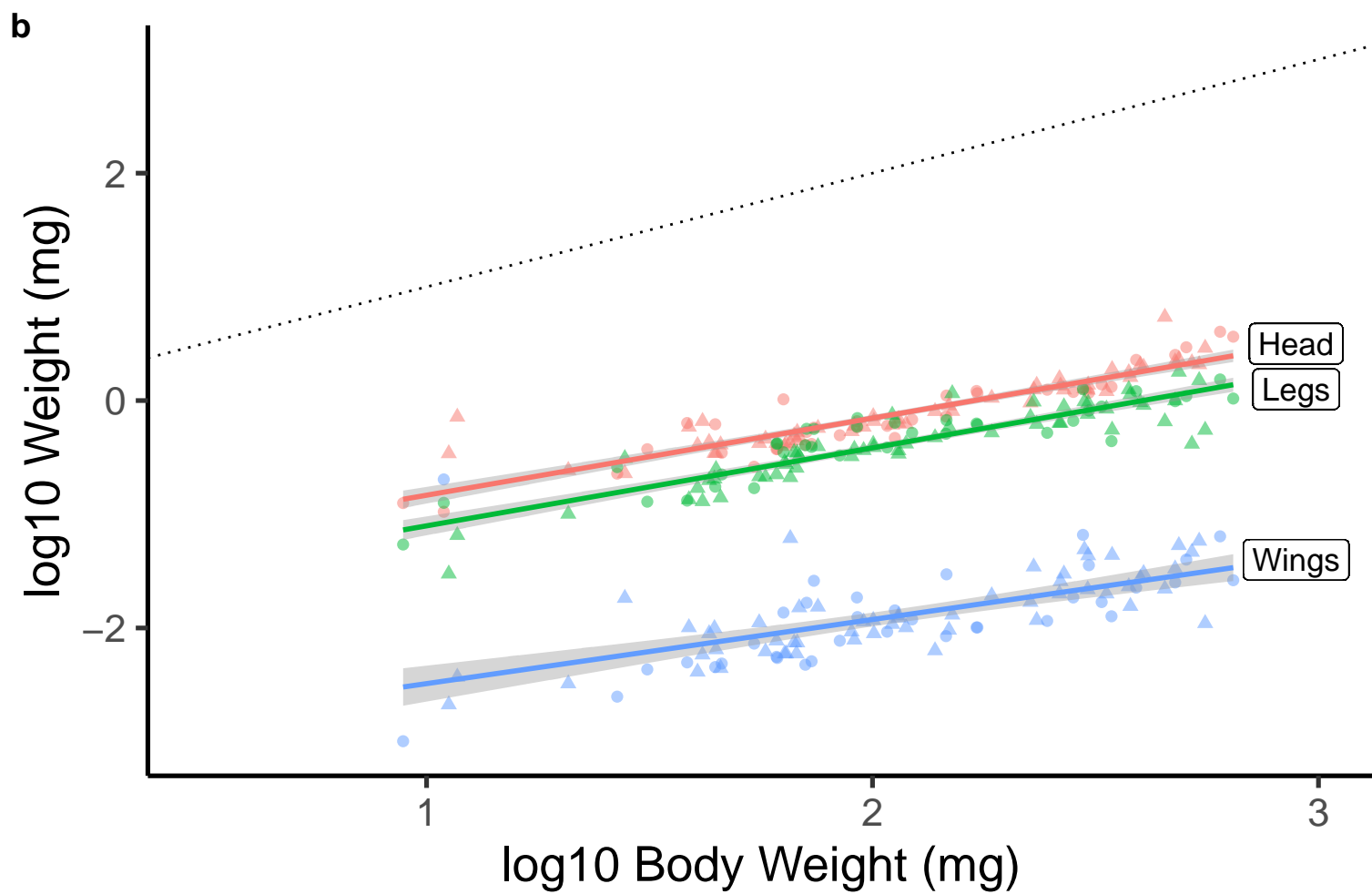

Supplement: Supplemental Information 1 — In both panels, the dotted line depicts the 1:1 line. The top panel (A) depicts thoraces and abdomens, with trendlines and 95% confidence intervals plotted from OLS regression models that included the effects of sex, additively. (B) Depiction of the scaling of heads, legs and wings with trendlines and 95% confidence intervals plotted from OLS regression models. In these tissues, there was no significant difference between males and females. [file peerj-08-8810-s001.pdf]
