# Supplementary material for: Within-host competition drives energy allocation trade-offs in an insect parasitoid
Source: PeerJ. 2020 Apr 21;8:e8810. doi: 10.7717/peerj.8810 (PMC7182028; doi:10.7717/peerj.8810)
Supplement: Supplemental Information 2 [file peerj-08-8810-s002.docx]

**Supplementary Table 1.** Model selection for relative weight investment in different body structures for *Drino rhoeo*

| **Body Segment** | **Model*** | **d.f.** | **F** | **p** | **R^2^** | **AIC** |
| --- | --- | --- | --- | --- | --- | --- |
| *Head* | ~ body weight | 1, 90 | 41.55 | < 0.0001 | 0.31 | -422 |
|  | ~ body weight + sex | 2, 89 | 20.82 | < 0.0001 | 0.30 | -420 |
|  | **~ poly(body weight 2)** | **2, 89** | **51.80** | **< 0.0001** | **0.53** | **-456** |
|  | ~ poly(body weight, 2) + sex | 3,88 | 35.02 | <0.0001 | 0.53 | -455 |
| *Thorax* | ~ body weight | 1, 90 | 0.62 | 0.4347 | 0.01 | -215 |
|  | ~ body weight + sex | 2, 89 | 24.40 | < 0.0001 | 0.34 | -252 |
|  | ~ poly(body weight, 2) | 2, 89 | 13.42 | < 0.0001 | 0.21 | -236 |
|  | **~ poly(body weight, 2) + sex** | **3, 88** | **35.98** | **< 0.0001** | **0.54** | **-284** |
| *Abdomen* | ~ body weight | 1, 90 | 33.74 | < 0.0001 | 0.26 | -241 |
|  | **~ body weight + sex** | **2, 89** | **65.18** | **< 0.0001** | **0.59** | **-292** |
|  | ~ poly(body weight, 2) | 2,89 | 24.08 | < 0.0001 | 0.35 | -249 |
|  | ~ body weight * sex | 3, 88 | 42.96 | < 0.0001 | 0.58 | -291 |
| *Wings* | **~ body weight** | **1, 90** | **9.79** | **0.0024** | **0.09** | **-489** |
|  | ~ body weight + Sex | 2, 89 | 5.17 | 0.0075 | 0.08 | -488 |
| *Legs* | ~ body weight | 1, 90 | 81.48 | <0.0001 | 0.47 | -531 |
|  | ~ body weight + sex | 2, 89 | 46.48 | <0.0001 | 0.50 | -535 |
|  | **~ body weight * sex** | **3, 88** | **32.73** | **<0.0001** | **0.51** | **-537** |

* All models described model the weight-normalized (weight of body structure/total fly weight) as the response variable. Poly(x,y) refers to a polynomial transformation of the variable, here it is always 2^nd^ order. Bold rows indicate the model we chose to best represent the relationship.
